# Improved diagnosis of *Trichuris trichiura* by using a bead-beating procedure on ethanol preserved stool samples prior to DNA isolation and the performance of multiplex real-time PCR for intestinal parasites

**DOI:** 10.1017/S0031182017000129

**Published:** 2017-03-14

**Authors:** MARIA M. M. KAISAR, ERIC A. T. BRIENEN, YENNY DJUARDI, ERLIYANI SARTONO, MARIA YAZDANBAKHSH, JACO J. VERWEIJ, TANIAWATI SUPALI, LISETTE VAN LIESHOUT

**Affiliations:** 1 Department of Parasitology, Faculty of Medicine, Universitas Indonesia, Jakarta, Indonesia; 2 Department of Parasitology, Leiden University Medical Center, Leiden, The Netherlands

**Keywords:** *Trichuris trichiura*, intestinal parasite, bead-beating, sample preparation, real-time PCR

## Abstract

For the majority of intestinal parasites, real-time PCR-based diagnosis outperforms microscopy. However, the data for *Trichuris trichiura* have been less convincing and most comparative studies have been performed in populations with low prevalence. This study aims to improve detection of *T. trichuria* DNA in human stool by evaluating four sample preparation methods. Faecal samples (*n* = 60) were collected at Flores island, Indonesia and examined by microscopy. Aliquots were taken and a bead-beating procedure was used both on directly frozen stool and on material preserved with 96% ethanol. PCR on frozen samples showed 40% to be positive for *T. trichiura*, compared with 45% positive by microscopy. The percentage positive increased when using ethanol preservation (45·0%), bead-beating (51·7%) and a combination (55·0%) and all three methods showed significantly higher DNA loads. The various procedures had a less pronounced effect on the PCR results of nine other parasite targets tested. Most prevalent were *Ascaris lumbricoides* (≈60%), *Necator americanus* (≈60%), *Dientamoeba fragilis* (≈50%) and *Giardia lamblia* (≈12%). To validate the practicality of the procedure, bead-beating was applied in a population-based survey testing 910 stool samples. Findings confirmed bead-beating before DNA extraction to be a highly efficient procedure for the detection of *T. trichiura* DNA in stool.

## INTRODUCTION

An estimated 1·45 billion people globally are infected with at least one or more of the soil-transmitted helminths (STH). Within this group whipworm (*Trichuris trichiura)* infects about 450 million people, mostly school age children (Pullan *et al.* 2014). Although many cases show only mild symptoms or are even asymptomatic, trichuriasis still has significant health consequences. Anaemia and poor nutrition status, especially in children, are partially attributable to chronic *T. trichiura* infections, while heavy worm burdens can result in ulcerative colitis and rectal prolapse (Knopp *et al.* 2012).

Diagnosis of STH as well as other intestinal parasites has always relied on the classical microscopic examination of stool samples as it is relatively simple to perform and does not require expensive laboratory equipment. However, microscopy is highly observer dependent, therefore lacking the opportunity to perform sufficient quality control. Moreover, the limited sensitivity has relevant consequences when monitoring the impact of mass drug administration as light infections are easily missed, hence population-based cure rates are overestimated (van Lieshout & Yazdanbakhsh, 2013; Knopp *et al.* 2014). The widely used Kato-smear-based stool examination for the detection of helminth eggs will miss most of the low-intensity infections and therefore specifically lacks sensitivity when approaching the end of the elimination phase in a control setting (Barda *et al.* 2015; Utzinger *et al.* 2015).

As an alternative to microscopy, DNA-based diagnostics have proved for over a decade to out-perform microscopy in the detection of gastro-intestinal parasites (Verweij, 2014). Real-time polymerase chain reaction (PCR) has been shown to be more specific and sensitive than direct parasitological techniques and its (semi) quantitative output also reflects the amount of parasite DNA present (Knopp *et al.* 2014; Verweij, 2014; Easton *et al.* 2016). Moreover, within a multiplex format different parasite targets can be detected in a single procedure. An advantage of DNA-based detection methods is the fact that stool samples when directly mixed with a preservative like ethanol, can be stored without immediate need of a cold chain (ten Hove *et al.* 2008). Therefore it is relatively simple to collect samples in remote rural areas and transport them to a central laboratory for further analysis without compromising the quality of the targeted DNA.

Nevertheless, despite its high-throughput screening potentials multiplex real-time PCR has not yet replaced microscopy for the diagnosis of STH in large-scale epidemiological surveys. Undoubtedly this can be explained by a range of logistical and financial challenges, which might be faced when implementing DNA detection-based diagnostics in a low resource laboratory setting. But an additional explanation has been the lack of a more efficient, though simple-to-use procedure to detect *T. trichiura* DNA in human stool samples within a multiplex or multi-parallel real-time PCR context. The most likely explanation for the relatively poor performance of the *T. trichiura* PCR, as seen in several studies, seems to be the robustness of *T. trichiura* eggs, hampering optimal DNA isolation (Verweij & Stensvold, 2014). Further improvement of the DNA isolation steps is therefore important, because as long as not each of the target helminth species can be diagnosed by the highly sensitive DNA detection-based methodology in combination with a relatively simple-to-use uniform sample-processing procedure, complementary microscopy examination of stool samples will remain indispensable (Wiria *et al.* 2013; Cimino *et al.* 2015; Gordon *et al.* 2015; Meurs *et al.* 2015).

To optimize DNA extraction for the detection of *T. trichiura*, several studies have suggested a supplementary bead-beating step proceeding a standard DNA extraction protocol (Andersen *et al.* 2013; Demeler *et al.* 2013; Liu *et al.* 2013; Platts-Mills *et al.* 2014; Easton *et al.* 2016). But only one short publication systematically evaluated the effect of bead-beating in relation to the microscopy outcome by comparing different procedures in a stool samples spiked with different concentrations of *T. trichiura* eggs. In this study, bead-beating actually did not result in a higher sensitivity of the *T. trichiura* PCR (Andersen *et al.* 2013).

Besides bead-beating, additional heating and centrifugation steps have been introduced specifically to enable efficient extraction of *T. trichiura* DNA (Mejia *et al.* 2013). Notably, nearly all studies applying these additional procedure showed a PCR-based prevalence of *T. trichiura* below 3%, which obscures the actual beneficial effects of these (Mejia *et al.* 2013; Cimino *et al.* 2015; Easton *et al.* 2016; Llewellyn *et al.* 2016).

The aim of the current study is to evaluate different methods to improve the detection of *T. trichuria* DNA in human stool. We therefore compared the effect of a bead-beating procedure prior to DNA extraction in stool samples with and without ethanol preservation. Stool samples were collected at Nangapanda village, situated in an area highly endemic for STH in Indonesia (Wiria *et al.* 2013). Samples were tested by multiplex real-time PCR for the presence of *T. trichiura* DNA as well as for nine other intestinal parasite targets and findings were compared with microscopy. In addition, to validate practical aspects of the procedure, bead-beating followed by *T. trichuria* DNA detection was applied in a large-scale population-based survey before and after intense anthelmintic treatment.

## MATERIALS AND METHODS

### Study design and sample collection

Stool samples were obtained from a multidisciplinary community research project named ‘Improving the health quality based on health education in Nangapanda sub-district, East Nusa Tenggara’, from the University of Indonesia. The study was approved by the ethics committee at the Faculty of Medicine, University of Indonesia with ethics number: 653/UN2.F1/ETIK/2014. In brief, this project focuses on the identification of factors that might contribute to food-borne diseases in young children living in Nangapanda sub-district, Ende, Flores Island, Indonesia. The study site has been selected partly based on the known high prevalence of STH (Wiria *et al.* 2013). The study started in January 2014. A total of 400 mothers were recruited together with their child, if within the age range of 1–5 years old. Participants gave written informed or parental consent. Each participant received an appropriate stool container and was asked to provide their fecal sample the following morning. The first 60 stool samples of at least 3 g were included. This sample size was based on a convenience sample that was limited by budget and personnel. The 60 samples originated from 38 mothers, ranging in age from 20 to 47 years (median 34 years) and 22 children (median age 3 years).

### Sample preparation and DNA isolation

**Fig. 1.**
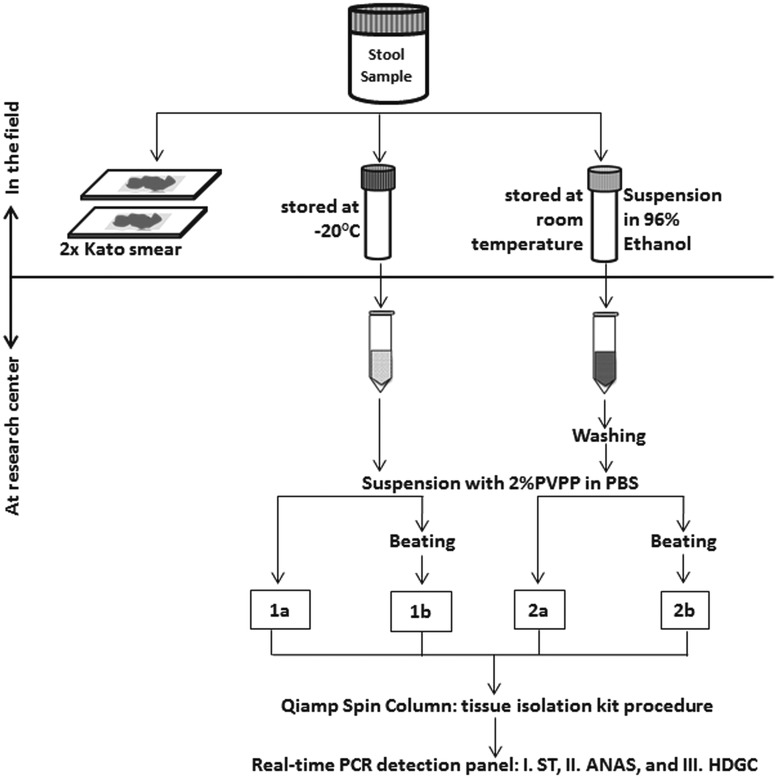
Flow-chart of the collection, preparations and measurements of 60 stool samples. Each preparation procedure is labelled as: 1a=C_PCR: PCR from directly frozen sample; 2a=E_PCR: PCR from ethanol preserved samples; 1b=B_PCR: PCR from bead-beating supplemented on frozen sample; 2b=E_B_PCR: PCR from bead-beating supplemented on ethanol-preserved samples. Real-time PCR detection: Panel I=ST (targetting *Schistosoma* sp. and *T. trichiura*); Panel II=ANAS (targetting *A. duodenale, N. americanus, A. lumbricoides* and *S. stercoralis*); Panel III=HDGC (targetting *E. histolytica, D. fragilis, G. lamblia* and *Cryptosporidium* spp.).

Figure 1 shows the sample preparation procedures used. Within a few hours after collection of the containers, two 25 mg Kato-smears were made and examined within 30–60 min by two independent microscopists for the presence of helminth eggs. In addition, two aliquots were taken from each stool sample. One aliquot of approximately 1–1·5 g was stored directly at −20 °C and kept frozen during transport. The other aliquot, equal to approximately 0·7 mL of volume, was mixed thoroughly with 2 mL of 96% ethanol by stirring with a wooden stick (ten Hove *et al.* 2008). These feces-ethanol solutions were initially stored at room temperature for around 4 weeks and thereafter stored and transported at 4 °C. Upon arrival at the Leiden University Medical Center (LUMC), the Netherlands, a custom-made automated liquid handling station (Hamilton, Bonaduz, Switzerland) was used for washing of samples, DNA isolation and the setup of the PCR plates. A washing step was applied to the preserved samples to remove the ethanol as described previously (ten Hove *et al.* 2008). Thereafter, both the washed samples and the frozen samples were suspended in 250 µL of PBS containing 2% polyvinylpolypyrrolidone (pvpp) (Sigma, Steinheim, Germany) (Verweij *et al.* 2009). From each suspension 200 µL was transferred to two deep-well plates (96 × 2·0 mL round, Nerbe Plus, Germany). From this point the following codes were used to label each of the four sample preparation procedures: C_PCR, which stands for controls, i.e. DNA extraction was performed on frozen samples without bead-beating; B_PCR, i.e. bead-beating was performed before DNA extraction on frozen samples; E_PCR, i.e. DNA extraction was performed on ethanol preserved samples; and E_B_PCR, bead-beating was performed before DNA extraction on ethanol preserved samples (Fig. 1). For the bead-beating procedure, 0·25 g of 0·8 mm garnet bead (Mobio US, SanBio Netherlands) was added to the B_PCR and E_B_PCR suspensions, followed by a beating process for 3 min at 1800 rotations per minute (rpm) using a homogenization instrument (Fastprep 96, MP Biomedical, Santa Ana California, USA). The choice of garnet bead was based on a small pilot study comparing five different bead types (see Supplementary Table S1). DNA extraction was performed using a spin column-based procedure as previously described (Wiria *et al.* 2010). In each sample, 10^3^ plaque-forming unit (PFU) mL^−1^ Phocin herpes virus-1 (PhHV-1) was included in the isolation lysis buffer, to serve as an internal control.

### Parasite DNA detection

Three different multiplex real-time PCR detection panels were used to detect and quantify parasite specific DNA of six helminth species and four species of intestinal protozoa. Panel I targets *Schistosoma* sp. and *T. trichiura*; Panel II targets *Ancylostoma duodenale, Necator americanus, Ascaris lumbricoides* and *Strongyloides stercoralis*; Panel III targets *Entamoeba histolytica, Dientamoeba fragilis, Giardia lamblia* and *Cryptosporidium* spp. The sequences of primers and probes and the setup of the PCR were based on published information and are also summarized in the Supplementary Table S2 and S3 (Niesters, 2002; Verweij *et al.*, 2003*a*, *b*; Verweij *et al.*, 2004; Verweij *et al.*, 2007*a*, *b*; Jothikumar *et al.*, 2008; Obeng *et al.*, 2008; Wiria *et al.*, 2010; Hamid *et al.*, 2011; Liu *et al.*, 2013). Amplification, detection and analysis were performed using the CFX real-time detection system (Bio-Rad laboratories). Negative and positive control samples were included in each PCR run. Cycle threshold (Ct) value results were analysed using Bio-Rad CFX software (Manager V3.1.1517·0823). The Ct-value represents the amplification cycle in which the level of fluorescent signal exceeds the background fluorescence (10^2^ Relative Fluorescents Units), reflecting the parasite-specific DNA load in the sample tested. The amplification of individual samples was considered to be hampered by inhibitory factors if the expected Ct-value of 31 in the PhHV-specific PCR was increased by more than 3·3 cycles. The PhHV PCR showed no significant reduction in Ct value as a result of the newly introduced sample preparation procedures. For each parasite-specific target, DNA loads were arbitrarily categorized into the following intensity groups: low (35 ⩽ Ct < 50), moderate (30 ⩽ Ct < 35) and high (Ct < 30) (Pillay *et al.* 2014).

### Application stage

An additional set of 910 stool samples was used to validate the practicality of *T. trichiura* DNA detection, in particular for the purpose of large-scale population-based surveys. Details of the study design have been published in the study protocol (Tahapary *et al.* 2015). In brief, faecal samples were collected at Nangapanda, on Flores Island, Indonesia, from 455 adults, age 16–83 years old (median 45 years), before and after a 1-year period of three monthly household treatments on three consecutive days with a single dose of 400 mg albendazole. Following microscopic examination of duplicate 25 mg Kato-smears, an aliquot of each stool sample was frozen within 24 h after collection and transferred to the Netherlands for laboratory analysis. For logistical reasons, explained in the discussion paragraph, it was decided in this study not to use ethanol preservation of stool samples. DNA isolation and detection of *T. trichiura* DNA was performed as described above according to the B_ PCR procedure. Pre- and post-treatment samples were tested pairwise, blinded from microscopy data.

### Data management and statistical analysis

All collected data were exported to the SPSS 20·0 (IBM, Chicago, IL) for statistical analysis and to Graph Pad 6 for visualization. Negative samples were recoded into an arbitrary value, i.e. 0·5 for egg counts and Ct 50 for PCR. Microscopy results were expressed as eggs per gram (EPG) of stool. Descriptive analysis was used to characterise the outcome of each sample preparation procedure. The percentages of positives were compared by their 95% confidence intervals (95% CI). The Wilcoxon signed-rank test was used to analyse a difference in median Ct-value between C_PCR (DNA extraction performed on frozen samples without bead-beating) and each of the alternative preparation procedures. This analysis was performed on those samples positive for at least one of the two indicated procedures and only for those parasite targets with nine or more positive samples. The Spearman's rho (*ρ*) value was used to indicate the strength of correlation between egg output (EPG) and DNA load (Ct-value) for *T. trichiura* and *A. lumbricoides*, as only these two have species-specific microscopy as well as PCR data available. Samples negative for both procedures were excluded in the statistical analysis. The McNemar test was used to compare paired proportions of microscopy and PCR detected *T. trichiura* cases in the population-based survey. A *P*-value <0·05 was considered to be statistically significant.

## RESULTS

### Intestinal parasite detection

Table 1 shows parasite detection rates as determined by microscopy and real-time PCR following each of the four fecal sample preparation procedures. None of the samples were positive for *Schistosoma* sp., *S. stercoralis* or *E. histolytica.* Based on microscopy *Ascaris lumbricoides* (60%) was the most prevalent STH found, followed by hookworm (46·7%) and *T. trichiura* (45·0%). The real-time PCR results from frozen samples (C_PCR) showed a lower percentage of *T. trichiura* positives (40·0%) compared to the Kato-smear (45·0%), while bead-beating (B_PCR) and the bead-beating on ethanol preserved samples (E_B_PCR) resulted in higher detection rates compared to microscopy, although the 95% CI did overlap. Using real-time PCR for the detection of hookworm, the number of positive cases ranged from 60·0 to 65·0%, with minor differences between the different sample preparation procedures. *Necator americanus* was found to be the dominant hookworm species. For *A. lumbricoides* the C_PCR procedure showed somewhat lower detection levels than microscopy, E_PCR, B_PCR and E_B_PCR, but none of the differences were significant. For intestinal protozoa the most prevalent species was *D. fragilis* (55·0%) followed by *G. lamblia* (16·7%) and for both species the highest detection rates were found with the E_PCR procedure.

**Table 1.** Number and percentage of parasite-positive cases detected either by microscopy or real-time PCR in 60 Indonesian stool samples

Parasite	No. of positive (%) [95% CI]				
	Kato smear	C_PCR	E_PCR	B_PCR	E_B_PCR
*Schistosoma* sp.	0	0	0	0	0
*T. trichiura*	27 (45·0) [32–58]	24 (40·0) [27–53]	27 (45·0) [32–58]	31 (51·7) [39–65]	33 (55·0) [42–68]
Hookworm	28 (46·7) [34–60]	37 (61·7) [49–74]	39 (65·0) [53–77]	36 (60·0) [47–73]	38 (63·3) [51–76]
* A. duodenale*		4 (6·7) [3–16]	5 (8·3) [4–18]	4 (6·7) [3–16]	4 (6·7) [3–16]
* N. americanus*		36 (60·0) [47–73]	36 (60·0) [47–73]	34 (56·7) [44–70]	36 (60·0) [47–73]
*A. lumbricoides*	36 (60·0) [47–73]	34 (56·7) [44–70]	36 (60·0) [47–73]	36 (60·0) [47–73]	37 (61·7) [49–70]
*S. stercoralis*	NA	0	0	0	0
*E. histolytica*	NA	0	0	0	0
*D. fragilis*	NA	31 (51·7) [39–65]	33 (55·0) [42–68]	26 (43·3) [30–56]	29 (48·3) [35–61]
*G. lamblia*	NA	9 (15·0) [8–26]	10 (16·7) [9–28]	6 (10·0) [5–20]	7 (11·7) [6–22]
*Cryptosporidium* spp.	NA	0	2 (3·3) [1–11]	0	1 (1·7) [0–9]

CI, Confidence Interval; NA, not applicable

The number of hookworm PCR from each preparation method were calculated based on *A. duodenale* plus *N. americanus* positives

### Stool parasite DNA load

**Fig. 2.**
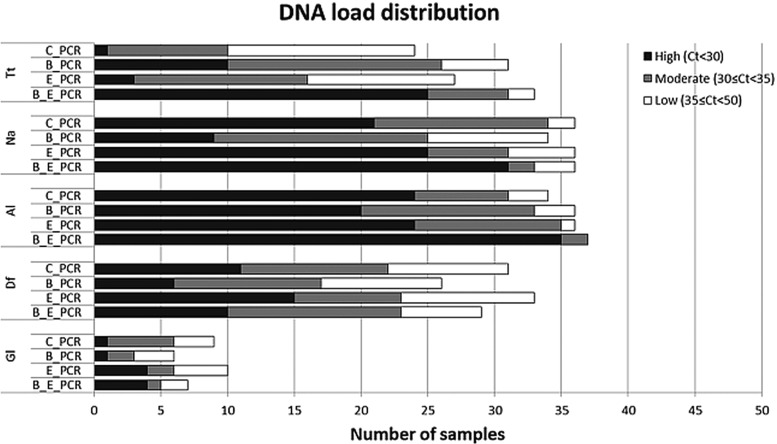
DNA load distribution of five most prominent intestinal parasites using four different preparation procedures on 60 stool samples. PCR results following the sample preparation procedure: C_PCR = directly frozen sample; E_PCR = ethanol-preserved sample; B_PCR = bead-beating supplemented on frozen sample; E_B_PCR = bead-beating supplemented on ethanol-preserved samples. Tt, *T. trichiura;* Na, *N. americanus*; Al, *A. lumbricoides*; Gl, *G. lamblia*; Df, *D. fragilis*.

Figure 2 displays the distribution of the PCR Ct-values for the five most common parasite targets for each of the sample preparation procedure, while Table 2 shows the comparison between the median Ct values. For *T. trichiura*, the C_PCR procedure showed low DNA loads (Ct ⩾ 35) in the majority of 24 PCR-positive samples. High DNA loads (Ct < 30) were detected in 10 of 31 PCR positives (32·3%) after bead-beating on frozen samples (B_PCR) and in 20 of 33 PCR positives (75·8%) after bead-beating on ethanol preserved samples (E_B_PCR). All three modified sample preparation procedures showed significant higher *T. trichiura* DNA levels compared with the control procedure (Table 2).

**Table 2.** Comparison of median cycle threshold (Ct) values between sample preparations procedures for the detected intestinal parasites

Species	Ct C_PCR *vs* E_PCR		*N*	*P*-value	Ct C_PCR *vs* B_PCR		*N*	*P*-value	Ct C_PCR *vs* E_B_PCR		*N*	*P* value
*T. trichiura*	35·35	33·82	29	**	36·29	32·02	34	***	36·38	28·53	35	***
*N. americanus*	29·30	28·75	37	NS	28·86	32·65	36	***	29·30	26·53	37	***
*A. lumbricoides*	28·11	28·95	36	NS	28·11	29·55	36	NS	28·21	24·77	37	***
*D. fragilis*	32·16	30·21	33	***	32·16	34·26	31	***	32·16	31·53	32	NS
*G. lamblia*	34·40	34·33	10	NS	34·23	35·87	9	**	34·40	36·49	10	NS

NS, not significant.

*P* value: *<0·05; **<0·01; ***<0·001.

Inclusion criteria: If result from one of the indicated procedure was positive. Analysis was done using Wilcoxon matched-pair rank test. C_PCR=PCR resulted from directly frozen sample; E_PCR=PCR resulted from ethanol preserved samples; B_PCR=PCR resulted from bead-beating supplemented on frozen sample; E_B_PCR=PCR resulted from bead-beating supplemented on ethanol-preserved samples.

For *N. americanus* most PCR positive samples were categorized as having moderate to high DNA loads (Ct < 35) in all four sample preparation procedures (Fig. 2). The bead-beating procedure on ethanol preserved samples (E_B_PCR) resulted in significantly higher *N. americanus* DNA levels compared to the controls. In contrast, when the bead-beating procedure was applied on frozen samples (B_PCR) *N. americanus* DNA levels were significantly lower compared with the controls (Table 2).

DNA loads in *A. lumbricoides* PCR-positive samples were mainly categorized as high (Fig. 2). This was most prominent in the E_B_PCR group with 35 of 37 PCR-positive samples (95·0%) showing a Ct below 30. The *A. lumbricoides* DNA load was significantly higher when comparing the E_B_PCR procedure with the control procedure, while no difference was seen for the two other sample preparation procedures (Table 2).

Using the bead-beating procedure (B_PCR) both the *D. fragilis* PCR and the *G. lamblia* PCR showed lower numbers of PCR-positive samples, as well as reduced DNA loads compared with the frozen controls (Fig. 2, Table 2). The same trend was seen when samples were preserved in ethanol.

**Fig. 3.**
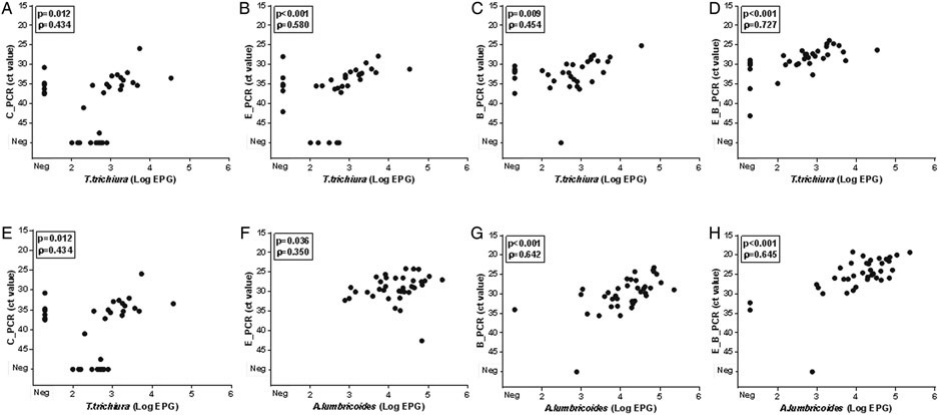
Association between egg output (Log EPG) and DNA load (Ct value) for *T. trichiura* (A–D) and *A. lumbricoide* (E–H). PCR results following the sample preparation procedure: C_PCR = directly frozen sample; E_PCR = ethanol-preserved sample; B_PCR = bead-beating supplemented on frozen sample; E_B_PCR = bead-beating supplemented on ethanol-preserved samples. Samples negative for both microscopy and real-time PCR were excluded in the statistical analysis.

Figure 3 shows the association between parasite DNA levels, represented by PCR Ct-value, and faecal egg count for *T. trichiura* and *A. lumbricoides. Trichuris trichiura* egg counts ranged from 60 to 33·740 epg (median 780 epg) and *A. lumbricoides* egg counts ranged from 760 to 226·520 epg (median 19·330 epg). For each of the four sample preparation procedures a positive correlation was found. For *T. trichiura*, the highest correlation coefficients were seen with the E_PCR procedure (Fig. 2B; *ρ* = 0·597, *n* = 32, *P* < 0·001) and the E_B_PCR procedure (Fig. 2D; *ρ* = 0·727, *n* = 33, *P* < 0·001). For *A. lumbricoides*, the highest correlation coefficients were seen when using the B_PCR procedure (Fig. 2G; *ρ* = 0·642, *n* = 37, *P* < 0·001) and the E_B_PCR procedure (Fig. 2H; *ρ* = 0·645, *n* = 38, *P* < 0·001).

### Application in an epidemiological survey

**Fig. 4.**
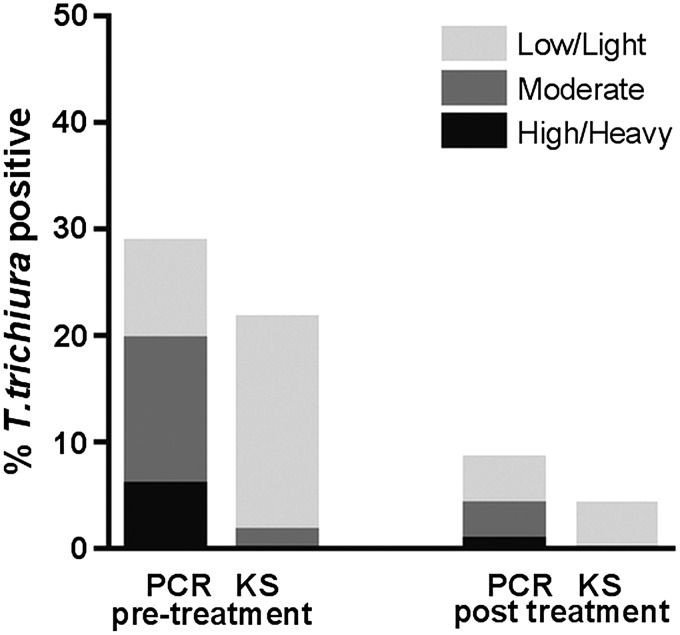
Prevalence and intensity of *T. trichiura* detected by PCR and Kato smear (KS) in 455 individuals before and 1 year after intense albendazole treatment. Ct values generated from real-time PCR were divided into three groups: high DNA load= Ct < 30, moderate DNA Load= 30 ⩽ Ct < 35 and low DNA load= 35 ⩽ Ct < 50. EPG is calculated from Kato smear detection and divided into three categories based on WHO criteria: heavy−=⩾10,000, moderate−= 1000–9999, and light-infection= 1–999.

Figure 4 shows a comparison between the prevalence and intensity of *T. trichiura* infection determined by microscopy and by PCR using the bead-beating procedure (B_PCR) in a large-scale population-based study. Based on microscopy 21·5% of this adult population (*n* = 455) was found to be positive for *T. trichiura* (range 20–5360 epg) prior to treatment, with 91 of the 98 positives showing <1000 epg and a median egg count of 100 epg. Following a year of intense anthelmintic treatment, eggs of *T. trichiura* were seen in 18 individuals (4·0%) with egg counts ranging from 20 to 400 epg (median 40 epg). Significantly more cases were detected by PCR than by microscopy, both before (28·8%) and after (8·4%) repeated rounds of albendazole treatment (*P* = 0·001).

## DISCUSSION

Molecular methods like real-time PCR are increasingly used in the diagnosis of intestinal helminths (Verweij and Stensvold, 2014; O'Connell and Nutman, 2016). However, large-scale application of DNA detection procedures seems to be impaired, partly by the fact that the standard DNA extraction methods are not sufficient to release DNA from *T. trichiura* eggs (Demeler *et al.* 2013). The present study aimed to identify a simple-to-use sample preparation procedure which could replace our previous standard method of stool DNA isolation, thereby increasing the PCR detection rate of *T. trichiura*-positive cases without uniformly changing the outcome of PCRs targeting other stool parasites.

In the pre-phase of the study, several mechanical procedures (e.g. extensive heating, vortexing, blending and sonication), chemical procedures (e.g. alkaline supplementation, adding of lyticase, achromopeptidase or a higher amount of proteinase K) and a number of combinations were evaluated, to see which method performed best to enhance the release of DNA from *T. trichiura* eggs, but none of them was very successful (data not shown). Better results were seen by the introduction of a bead-beating step, which facilitates the breakdown of the proteinaceous cellular wall, thereby making the DNA accessible (Verollet, 2008; Demeler *et al.* 2013). Several beads types of different manufacturers were compared and although tested in a small pilot only; differences were seen between the type of beads used (Supplementary Table S1). Based on these findings garnet beads were selected for the further evaluation of the procedures.

Although the use of bead-beating to facilitate helminth DNA isolation has been mentioned in several publications, details of the performed procedures are mostly limited or even lacking. At most a comparison is made with the number of cases detected by PCR *vs* the number of cases detected by microscopy (Liu *et al.*, 2013, 2016; Taniuchi *et al.*, 2013; Platts-Mills *et al.*, 2014; Easton *et al.*, 2016). For example, in a study performed on 400 unpreserved stool samples collected from 13-month-old children in Ecuador, 3% of the samples were positive for *T. trichiura* by PCR compared to 0·75% positive cases by microscopy (Mejia *et al.* 2013). In this study, additional heating and centrifugation steps were introduced specifically to enable efficient extraction of *T. trichiura* DNA, but no details have been given and the overall low prevalence of *T. trichiura* makes it difficult to judge the importance of these additional procedures. Moreover, no adequate internal control was implemented to assess potential inhibition during DNA isolation and detection (Mejia *et al.* 2013).

To our knowledge, Andersen and colleagues are the only group to have published details on the effect of adding beads on the detection of *T. trichiura* DNA by real-time PCR (Andersen *et al.* 2013). In this study, three different types of beads, namely glass, garnet and zirconium, were evaluated. Bead-beating was compared with vortexing in the presence of beads. The authors concluded that glass beads were not very practical due to the increased risk of clotting of the tips and that none of the evaluated procedures increased the DNA yield from eggs of *T. trichiura* (Andersen *et al.* 2013). However, these findings have been based only on a stool sample artificially spiked with helminth eggs and on a single *T. trichiura*-positive clinical sample.

In the current study, 60 stool samples have been collected from a region in Indonesia known to be highly endemic for STH. This high transmission level was confirmed by the detection of *T. trichiura* eggs in 45% of the stool samples after performing a duplicate 25 mg Kato-smear examination. However, egg excretion was not correspondingly high in this population with less than half of the positive samples showing more than 1000 epg. Despite the low intensity, this collection of stool samples was regarded as highly suitable for comparing different sample preparation procedures, noting that the majority of studies applying DNA detection-based diagnostic procedures work with a PCR-based prevalence of *T. trichiura* below 3% (Mejia *et al.* 2013; Cimino *et al.* 2015; Easton *et al.* 2016; Llewellyn *et al.* 2016). In addition, the presented collection of stool samples showed high levels of co-infections with other parasites, both helminths and protozoa, so the effect of different sample preparation procedures could be evaluated as well on DNA detection of *N. americanus, A. lumbricoides, D. fragilis* and *G. lamblia*.

Similarly to our previous, though unpublished, findings on stool samples which have been frozen only, the number of *T. trichiura* PCR-positive cases was lower than the number of microscopy-positive cases if no additional sample preparation procedures were applied. Indeed, without the addition of bead-beating the DNA yields of *T. trichiura* were generally low, with a minority of samples showing a Ct-value below 30. This in contrast to the effect of bead-beating, in particular on ethanol preserved samples, which resulted in the detection of additional helminth-positive cases. Naturally the yield of *T. trichiura* DNA was also found to be substantially higher. The actual mechanism explaining why the addition of ethanol preservation resulted in higher DNA loads detected is not completely clear. The opposite might have been expected as the addition of ethanol requires a washing step, which discharges any free DNA present in the sample. The beneficial effect of combining bead-beating with ethanol preservation was found to be most distinct for the helminth species, in particular for *T. trichiura*, and not for the protozoa, suggesting the effect to be parasite specific.

Ethanol is widely used as a preservative of biological samples, including fecal material, when nucleic acid-based testing is needed and it was introduced several years ago to facilitate molecular diagnosis of helminth infections in remote populations where no cold-chain is available (ten Hove *et al.* 2008). Despite the evident advantages of ethanol preservation shown in this study, we do not advocate the routine use of ethanol for all epidemiological studies dealing with stool collection. Three disadvantages of ethanol preservation have led to this new policy. Firstly, based on our experience in a number of population-based surveys, we noticed that the mixing of stool and ethanol in a field setting can be a critical process. Not only the ratio between the fecal material and the preservative is crucial, but the mixing must be thorough otherwise proper preservation will not take place. In case no vortex is available, this should be done by thoroughly stirring with a wooden stick. Secondly, the tubes should be well sealed to prevent leakage during transportation. Finally, the addition of a preservative will make the DNA extraction procedure more laborious and therefore more expensive as the ethanol has to be washed away before actual DNA extraction can proceed. This washing is an additional step in the sample handling procedure which increases the overall risk of human error, in particular if no automated sampling system is available.

In the past, we have encountered errors resulting from each of the three points mentioned above, resulting in the loss of samples for molecular diagnosis, despite detailed instructions to field teams. In the case that no aliquots have been frozen and no supplementary microscopy has been performed, the loss of samples due to inappropriate preservation could be devastating. For these reasons we commonly advise our collaborators to directly freeze the samples whenever a proper cold-chain can be guaranteed or otherwise be extremely cautious concerning the point discussed above.

Because of the these practical limitations of ethanol preservation, direct freezing of stool samples has been applied in a recently performed survey on Flores Island, Indonesia, studying the effect of three monthly household treatments with albendazole (Tahapary *et al.* 2015). Based on microscopy only, the prevalence of *T. trichiura* was found to be 21·5% at baseline, and with a median egg count of 100 epg, intensity of infection was already low before the intervention started. The relatively narrow range in egg excretion in this population probably explains why no correlation was found between the intensity of infection based on microscopy and the DNA load as determined by real-time PCR (data not shown). Following a year of intense treatment, a clear reduction was seen in the number of *T. trichiura*-positive cases, both based on microscopy and on DNA detection. Still, around twice as many *T. trichiura*-positives cases were detected by PCR than by microscopy, confirming the higher sensitivity of molecular diagnosis assuming appropriate DNA isolation procedures have been performed (Verweij, 2014; van Lieshout and Roestenberg, 2015).

Our results overall illustrate that relatively minor differences in sample handling procedures can influence the output of real-time PCR. Consequently, comparison of studies, in particular when evaluating intensity of infections, should be interpreted with great care, especially if molecular testing has been performed in different laboratories or procedures have been changed over-time. The use of a standard curve, by adding genomic DNA at different concentrations or plasmids constructed to match the same target sequence, gives the impression that PCR yields standard results. But this does not allow for the effect of different isolation procedures. More progress is expected from defining an internationally recognized standard based on a fixed number of eggs per volume of stool.

In conclusion, the present findings confirm that a bead-beating procedure prior to DNA extraction increases the *T. trichiura* DNA yield in human faecal samples. Moreover, in combination with ethanol preservation this effect is most pronounced and also improves the detection rate of *N. americanus* and *A. lumbricoides*. Although ethanol preservation in itself has a positive effect, it also imposes several practical limitation. The effect of bead-beating on the detection of intestinal protozoa does not seem to be as uniform. Therefore bead-beating should be implemented with some caution, in particular when detecting *D. fragilis* in non-preserved samples. The general finding that sample handling procedures significantly influence detected DNA yield illustrates the potential impact of well-defined sample handling procedures and confirms the importance of standardisation. Exchange of proficiency panels between centralised reference laboratories seems an essential step in further harmonisation of molecular diagnosis of intestinal helminths.

## Supplementary material

For supplementary material accompanying this paper visit http://dx.doi.org/10.1017/S0031182017000129. click here to view supplementary material
