# Supplementary material for: Improved diagnosis of Trichuris trichiura by using a bead-beating procedure on ethanol preserved stool samples prior to DNA isolation and the performance of multiplex real-time PCR for intestinal parasites
Source: Parasitology. 2017 Mar 14;144(7):965–74. doi: 10.1017/S0031182017000129 (PMC5471844; doi:10.1017/S0031182017000129)
Supplement: Supplementary file 1 [file S0031182017000129sup001.docx]

**Improved diagnosis of *Trichuris trichiura* by using a bead-beating procedure on ethanol preserved stool samples prior to DNA isolation and the performance of multiplex real-time PCR for intestinal parasites.**

MARIA M. M. KAISAR, ERIC A.T. BRIENEN, YENNY DJUARDI, ERLIYANI SARTONO, MARIA YAZDANBAKHSH, JACO J. VERWEIJ, TANIAWATI SUPALI, LISETTE VAN LIESHOUT**^*^**

*Corresponding author. Department of Parasitology, Leiden University Medical Center, Albinusdreef 2 (zone L4-Q) 2300 RC, Leiden, The Netherlands. E-mail: [lvanlieshout@lumc.nl](mailto:lvanlieshout@lumc.nl)

**Supplementary Information**

**Table S1.** The comparison of cycle threshold (Ct) -values for different bead types tested in bead-beating procedure for *T. trichiura* detection

| Sample ID | Egg count* | Real-time PCR (Ct-value) | | | | | |
| --- | --- | --- | --- | --- | --- | --- | --- |
|  |  | C_PCR | Type of beads used in B_PCR** | | | | |
|  |  |  | Stainless Steel | Zirconium Oxide | Matrix A | Garnet | Matrix Y |
| A1284 | 61 | 34.18 | 27.13 | 24.69 | 25.14 | 26.58 | 26.40 |
| A1311 | 42 | 34.33 | 27.15 | 26.91 | 27.13 | 26.00 | 0 |
| D537 | 2 | 0 | 0 | 37.01 | 0 | 33.97 | 0 |
| D680 | 0 | 0 | 0 | 0 | 0 | 0 | 0 |
| H3404 | 162 | 37.06 | 32.48 | 34.98 | 31.52 | 29.15 | 35.03 |
| H4022 | 374 | 37.75 | 32.87 | 28.06 | 26.32 | 27.72 | 27.55 |

C_PCR=PCR resulted from directly frozen sample; B_PCR=PCR resulted from bead-beating supplemented on frozen sample.

* A single slide microscopy examination performed using formol-ether concentration procedure

** The sizes of the beads are the following: Stainless steel [nextadvance, NY] is 0.5 mm; Zirconium Oxide [nextadvance, NY] is 0.5 mm; Matrix A is a 0.7 mm Garnet Lysing matrix [MP Biomedicals]; Garnet is 0.8 mm bead [Mobio US, SanBio Netherlands]; Matrix Y are 0.5 mm Yttria-Stabilized Zirconium Oxide beads [MP Biomedicals]. The volume of bead used per sample was 1.072 cm^3^, this volume corresponds to 0.50 gram, 0.30 gram, 0.15 gram, 0.25 gram and 0.30 gram of beads which are, respectively, steel, zirconium oxide, matrix A, garnet and Matrix Y.

**Table S2.** Oligonucleotide primers and detection probes for real-time PCR for the simultaneous detection of intestinal helminth and protozoa

| Target organism | Oligo name | Oligonucleotide sequences | Reference |
| --- | --- | --- | --- |
| *Schistosoma* sp. | Ssp_ITS_48F | 5’- GGTCTAGATGACTTGATYGAGATGCT -3’ | (Obeng *et al.*, 2008) |
|  | Ssp_ITS_124R | 5’- TCCCGAGCGYGTATAATGTCATTA -3’ |  |
|  | Ssp_ITS_78T_FAM | FAM-5’- TGGGTTGTGCTCGAGTCGTGGC -3’-BHQ1 |  |
| *Trichuris trichiura* | Tt_283F | 5’- TTGAAACGACTTGCTCATCAACTT -3’ | (Liu *et al.*, 2013) |
|  | Tt_358R | 5’- CTGATTCTCCGTTAACCGTTGTC -3’ |  |
|  | Tt_308T_YY | Yakima Yellow-5’- CGATGGTACGCTACGTGCTTACCATGG -3’-BHQ1 |  |
| *Ancylostoma* sp. | Ad_125F | 5’- GAATGACAGCAAACTGCTTGTTG -3’ | (Hamid *et al.*, 2011; Verweij *et al.*, 2009; Wiria *et al.*, 2010) |
|  | Ad_195R | 5’- ATACTAGCCACTGCCGAAACGT -3’ |  |
|  | Ad_155_XS_TR | Texas red-5’- ATCGTTTACCGACTTTAG -3’BHQ2 |  |
| *Necator americanus* | Na_58F | 5’- CTGGTTTGTCGAACGGTACTTGC -3’ |  |
|  | Na_158R | 5’- ATAACAGCGTGCACATGTTGC -3’ |  |
|  | Na_81T_XS_FAM | FAM-5’- CTGTACTACGCATTGTATAC -3’-BHQ1 |  |
| *Ascaris lumbricoides* | Alum_96F | 5’- GTAATAGCAGTCGGCGGTTTCTT -3’ |  |
|  | Alum_183R | 5’- GCCCAACATGCCACCTATTC -3’ |  |
|  | Alum_124T_YY | Yakima Yellow-5’- TTGGCGGACAATTGCATGCGAT -3’-BHQ1 |  |
| *Strongyloides stercoralis* | Stro 18S-1530F | 5’- GAATTCCAAGTAAACGTAAGTCATTAGC -3’ |  |
|  | Stro 18S-1630R | 5’- TGCCTCTGCATATTGCTCAGTTC -3’ |  |
|  | Stro 18S-1586T | Quasar705-5’- ACACACCGGCCGTCGCTGC -3’-BHQ2 |  |
| *Entamoeba histolytica* | Ehd_F | 5’- ATTGTCGTGGCATCCTAACTCA -3’ | (Verweij *et al.*, 2003a) |
|  | Ehd_R | 5’- GCGGACGGCTCATTATAACA -3’ |  |
|  | Eh_18S_XS_YY | Yakima Yellow-5’- TCATTGAATGAATTGGCCATTT -3’-BHQ1 |  |
| *Dientamoeba fragilis* | Df_124F | 5’- CAACGGATGTCTTGGCTCTTTA -3’ | (Verweij *et al.*, 2007) |
|  | Df_221R | 5’- TGCATTCAAAGATCGAACTTATCAC -3’ |  |
|  | Df_172_XS_Quasar705 | Yakima Yellow-5’- CAATTCTAGCCGCTTAT -3’-BHQ1 |  |
| *Giardia lamblia* | Giardia_18S_99F | 5’- GACGGCTCAGGACAACGGTT -3’ | (Verweij *et al.*, 2004; Verweij *et al.*, 2003b) |
|  | Giardia_18S_125R | 5’- TTGCCAGCGGTGTCCG -3’ |  |
|  | Giardia_18S_FAM | FAM 5’- CCCGCGGCGGTCCCTGCTAG -3’-BHQ1 |  |
| *Cryptosporidium* spp. | Cr_spp_JVAF | 5’- ATG ACG GGT AAC GGG GAAT -3’ | (Jothikumar *et al.*, 2008) |
|  | Cr_spp_JVAR | 5’- CCA ATT ACA AAA CCA AAA AGT CC -3’ |  |
|  | Cr_spp_JVAP18S_TR | Texas Red 5’- CGC GCC TGC TGC CTT CCT TAG ATG -3’-BHQ2 |  |
| Phocin Herpes Virus (PhHV) | PhHV_267s | 5’- GGGCGAATCACAGATTGAAT\|C -3’ | (Niesters, 2002) |
|  | PhHV_337as | 5’- GCGGTTCCAAACGTACCAA -3’ |  |
|  | PhHV_305tq_Cy5 | Cy5-5’- TTTTTATGTGTCCGCCACCATCTGGATC -3’-BHQ2 |  |

**Table S3.** Mixtures composition in three real-time PCR panels used for intestinal parasites detection

| Panel I: ST | | |
| --- | --- | --- |
| Reagents: | Concentration: | 1 Sample: |
| H_2_0 |  | 2,025 |
| MgCl_2_ | 25 mM | 3,50 |
| BSA | 5 mg/ml | 0,50 |
| Primer Ssp -F | 25 µM | 0,20 |
| Primer Ssp -R | 25 µM | 0,20 |
| Probe Ssp -FAM | 10 µM | 0,125 |
| Primer Tt -F | 25 µM | 0,20 |
| Primer Tt -R | 25 µM | 0,20 |
| Probe Tt -YY | 10 µM | 0,125 |
| Primer PHHV -S | 25 µM | 0,15 |
| Primer PHHV -AS | 25 µM | 0,15 |
| Probe PHHV - Cy5 | 10 µM | 0,125 |
| HotStar Taq Master Mix |  | 12,50 |
| Total |  | 20,00 |
| Add 5 µl DNA to the mix |  |  |

| Panel II: ANAS | | |
| --- | --- | --- |
| Reagents : | Concentration: | 1 Sample: |
| H_2_0 |  | 2,025 |
| MgCl_2_ | 25 mM | 3,50 |
| BSA | 5 mg/ml | 0,50 |
| Primer Ad -F | 25 µM | 0,20 |
| Primer Ad -R | 25 µM | 0,20 |
| Probe Ad - TR | 10 µM | 0,25 |
| Primer Na -F | 25 µM | 0,20 |
| Primer Na -R | 25 µM | 0,20 |
| Probe Na - FAM | 10 µM | 0,125 |
| Primer Alum -F | 10 µM | 0,20 |
| Primer Alum -R | 10 µM | 0,20 |
| Probe Alum - YY | 10 µM | 0,125 |
| Primer Stro -F | 25 µM | 0,10 |
| Primer Stro -R | 25 µM | 0,10 |
| Probe Stro -Quasar 705 | 10 µM | 0,125 |
| Primer PHHV -S | 25 µM | 0,15 |
| Primer PHHV -AS | 25 µM | 0,15 |
| Probe PHHV - Cy5 | 10 µM | 0,125 |
| HotStar Taq Master Mix |  | 12,50 |
| Total |  | 20,00 |
| Add 5 µl DNA to the mix |  |  |

| Panel III: HDGC | | | | | |  |  |  |
| --- | --- | --- | --- | --- | --- | --- | --- | --- |
| Reagents: | Concentration: | | 1 Sample: | | | | | |
| H_2_0 | |  | | | 1,61 | | |  |
| MgCl_2_ | | 25 mM | | 3,50 | | |  |  |
| BSA | | 5 mg/ml | | 0,50 | | |  |  |
| Primer Ehd -F | | 25 µM | | 0,04 | | |  |  |
| Primer Ehd -R | | 25 µM | | 0,04 | | |  |  |
| Probe Eh - YY | | 10 µM | | 0,125 | | |  |  |
| Primer Df -F | | 25 µM | | 0,20 | | |  |  |
| Primer Df -R | | 25 µM | | 0,20 | | |  |  |
| Probe Df -Quasar705 | | 10 µM | | 0,25 | | |  |  |
| Primer Giardia -F | | 25 µM | | 0,06 | | |  |  |
| Primer Giardia -R | | 25 µM | | 0,06 | | |  |  |
| Probe Giardia -FAM | | 10 µM | | 0,125 | | |  |  |
| Primer Cr spp -F | | 25 µM | | 0,06 | | |  |  |
| Primer Cr spp -R | | 25 µM | | 0,06 | | |  |  |
| Probe Cr spp -TR | | 10 µM | | 0,25 | | |  |  |
| Primer PHHV - S | | 25 µM | | 0,15 | | |  |  |
| Primer PHHV -AS | | 25 µM | | 0,15 | | |  |  |
| Probe PHHV - Cy5 | | 10 µM | | 0,125 | | |  |  |
| HotStar Taq Master Mix | |  | | 12,50 | | |  |  |
| Total | |  | | 20,00 | | |  |  |
| Add 5 µl DNA to the mix | |  | |  | | |  |  |
